# Fluorodeoxyglucose Positron Emission Tomography Evaluation of Chronic Recurrent Multifocal Osteomyelitis

**DOI:** 10.7759/cureus.69735

**Published:** 2024-09-19

**Authors:** Ryosuke Hirota, Makoto Emori, Atsushi Teramoto

**Affiliations:** 1 Department of Orthopaedic Surgery, Sapporo Medical University, Sapporo, JPN

**Keywords:** bone tumor, chronic recurrent multifocal osteomyelitis, clinical case report, ewing's sarcoma, fdg-pet (fluorodeoxyglucose-positron emission tomography)

## Abstract

Chronic recurrent multifocal osteomyelitis (CRMO) is an autoinflammatory bone disease that predominantly affects children and adolescents. Currently, CRMO diagnoses are based on a combination of clinical, radiological, pathological, and longitudinal findings. However, distinguishing CRMO from malignant bone tumors using imaging is occasionally challenging. Fluorodeoxyglucose (FDG) positron emission tomography (PET) imaging characteristics in CRMO (specifically, the maximum standardized uptake value (SUVmax)) have been described previously. The lesions exhibited increased FDG uptake despite the benign nature of the disease; the SUVmax was notably high (4.90). These findings suggest that FDG/PET plays a limited role in the differential diagnosis of CRMO.

## Introduction

Chronic recurrent multifocal osteomyelitis (CRMO) is a rare autoinflammatory disorder that mainly affects children and adolescents, usually appearing within the first two decades of life [1]. It is characterized by recurrent episodes of bone pain linked with sterile inflammatory lesions that can occur in various parts of the body, particularly in long bones, clavicle, pelvis, and vertebrae [2]. The precise pathogenesis of CRMO remains unclear, though it is believed to involve genetic predisposition and environmental factors that trigger an abnormal immune response. Unlike infectious osteomyelitis, CRMO has no infectious agent, and its lesions are typically sterile, making diagnosis based solely on clinical presentation challenging.

Imaging studies, including whole-body magnetic resonance imaging (MRI) and fluorodeoxyglucose (FDG) positron emission tomography (PET), are crucial for diagnosing CRMO. MRI is highly sensitive for detecting both symptomatic and asymptomatic lesions. However, its findings can resemble those of other conditions, such as infections and malignancies. In these instances, FDG/PET can aid in distinguishing malignant from benign lesions by assessing metabolic activity, although inflammatory CRMO lesions may lead to false positives. This complexity underscores the pertinence of careful interpretation of imaging results.

We present a case of CRMO where FDG/PET findings were significant in the diagnostic process. This case is noteworthy because such detailed imaging findings are rarely documented, allowing for discussion on the utility and limitations of FDG/PET in differentiating CRMO from malignancies. Given the potential for incorrect results, we explore whether FDG/PET should be routinely used or reserved for cases where other diagnostic methods are inconclusive.

## Case presentation

**Figure 1:**
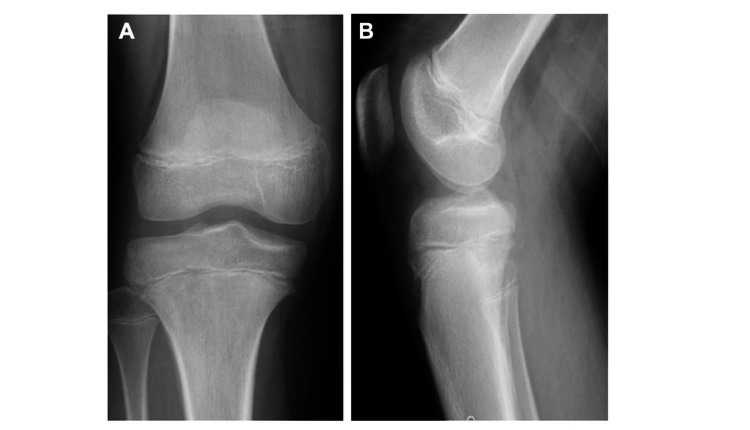
X-ray of the right knee: (A) Anteroposterior view and (B) lateral view. No significant bone destruction or sclerosis is observed.

A nine-year-old girl presented with progressively worsening pain in the proximal right lower leg, accompanied by redness and localized heat. Her white blood cell count (WBC) and C-reactive protein (CRP) level were 7.1 × 10^9 cells/L (neutrophils 62.0%, lymphocytes 34.0%, monocytes 4.0%) and 1.48 mg/dL (normal: <0.30 mg/dL), respectively. All other biochemical and serologic tests were normal. X-ray examination did not reveal any obvious bone destruction in either the tibia or fibula (Figure 1).

**Figure 2:**
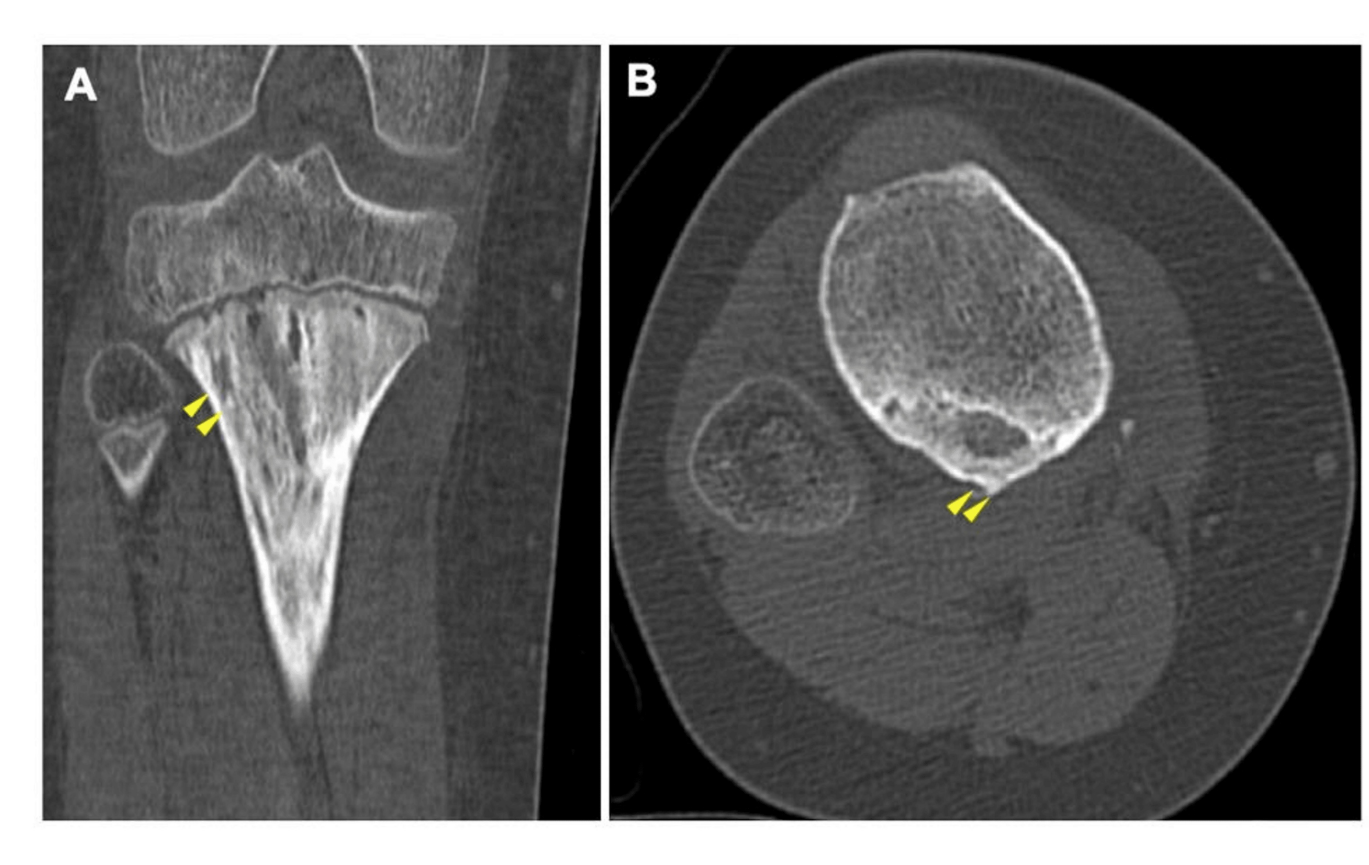
Computed tomography images of the right knee. (A) Coronal view and (B) axial view. Computed tomography images show bone destruction with some calcification in the proximal tibia (yellow arrow).

Computed tomography (CT) revealed bone destruction on the medial aspect of the proximal tibia with some calcification (Figure 2). 

**Figure 3:**
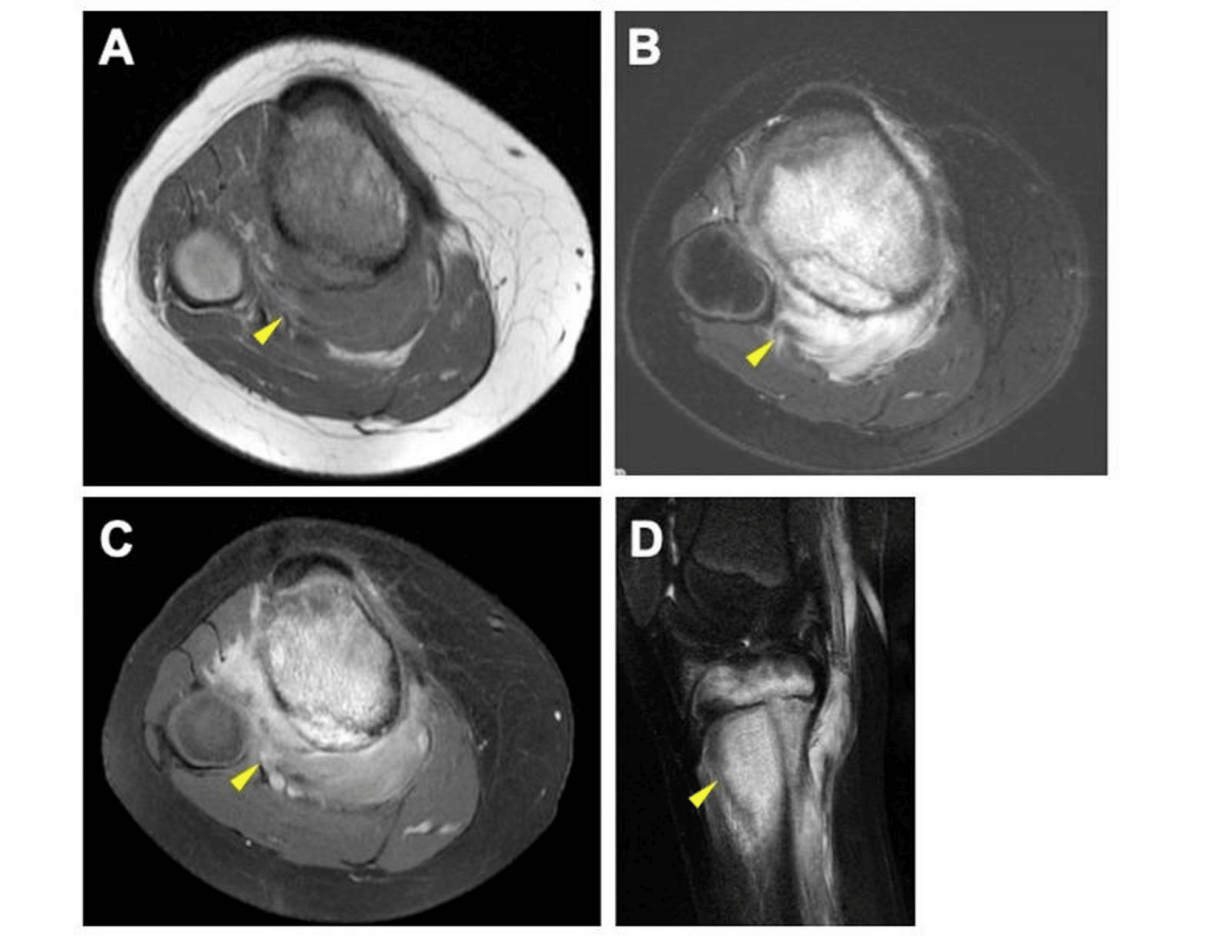
MRI of the proximal tibia. (A) Axial T1-weighted image, (B) axial T2-weighted image, (C) axial gadolinium-enhanced MRI, and (D) sagittal gadolinium-enhanced MRI Magnetic resonance imaging of the proximal tibia metaphysis shows a hypointense mass on a T1-weighted image (A) and a hyperintense mass on a T2-weighted image (B). The mass shows enhancement on a T1-weighted image after administering gadolinium (C) and (D) (yellow arrow).

MRI revealed a mass in the metaphyseal region of the proximal tibia, which appeared hypointense on T1-weighted imaging and hyperintense on T2-weighted imaging, with evidence of extraosseous extension (Figure 3).

**Figure 4:**
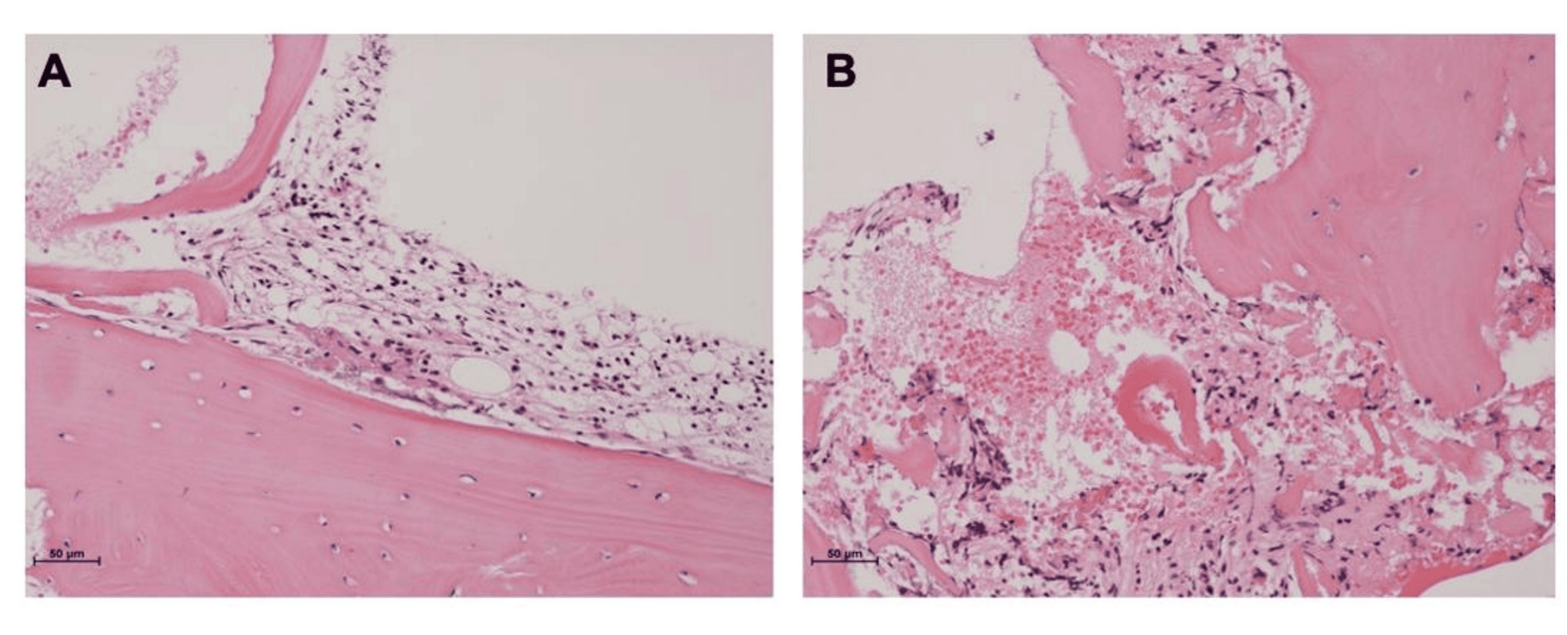
A histological analysis of the primary tumor reveals infiltrating inflammatory cells, proliferating collagen fibers, and no atypical cells (A and B).

All other biochemical and serologic tests were normal. Based on these hematological investigations and imaging findings, osteomyelitis was initially suspected. The patient was treated with antibiotics (cefcapene pivoxil [CFPN-PI]) for 20 days, but there was no improvement. Consequently, the possibility of malignant bone tumors, such as Ewing's sarcoma, was considered. An incisional biopsy was performed, and the histological analysis revealed infiltration of inflammatory cells, proliferation of collagen fibers, and the absence of atypical cells (Figure 4).

Bacterial cultures from the biopsy specimen were negative. The lesion was thus diagnosed as CRMO, leading to the discontinuation of antibiotics, and the patient was discharged two months later.

**Figure 5:**
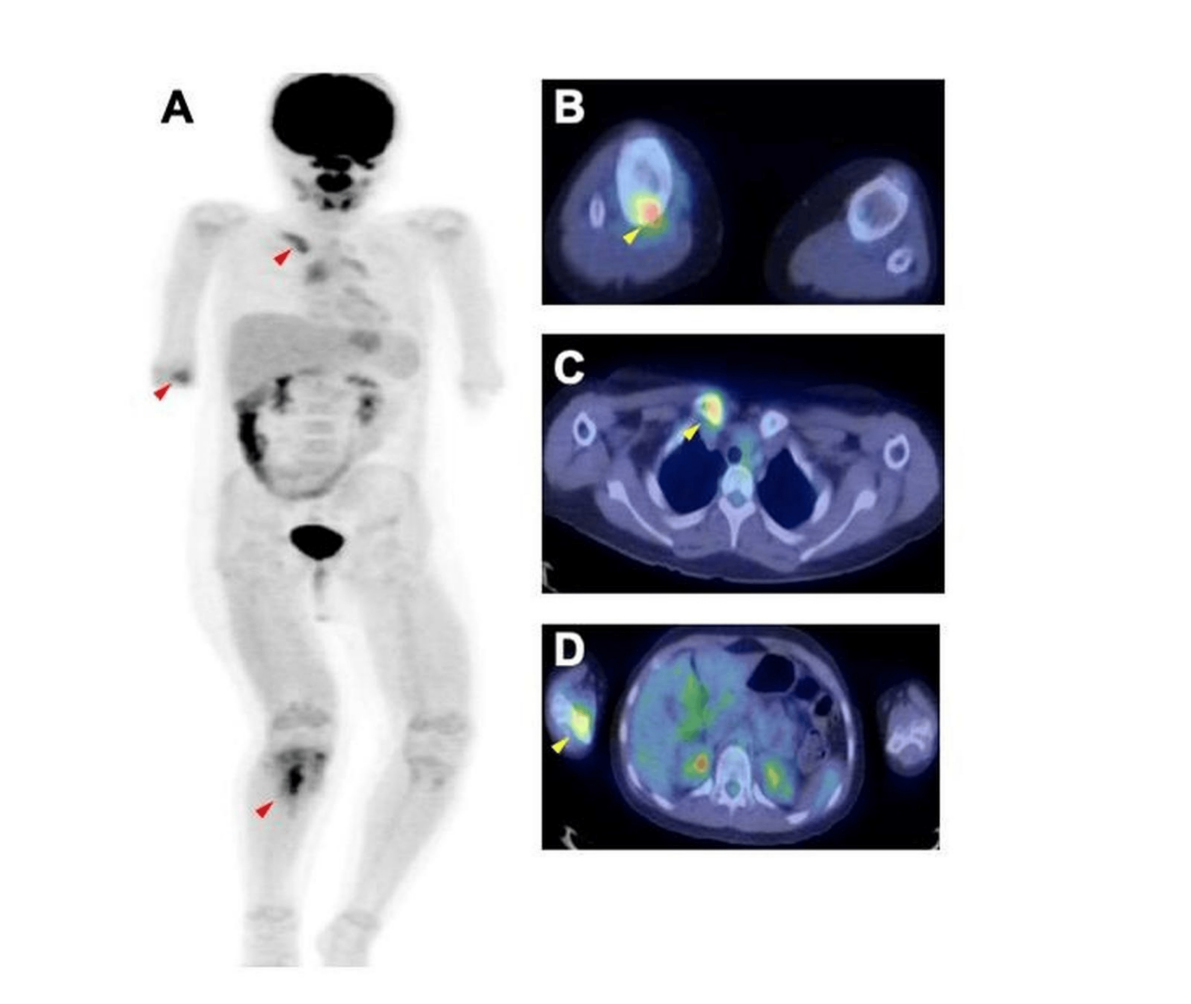
FDG/PET imaging was performed for a systemic search (A), which revealed increased uptake in the right proximal tibia (SUVmax 4.90) (B), right proximal clavicle (SUVmax 3.20) (C), and distal humerus (SUVmax 3.20) (D) (red and yellow arrow).

Four months post-discharge, she developed swelling and sharp pain in her clavicle and elbow. MRI of the clavicle showed findings consistent with those of the tibia. Given the potential for undetected malignant areas in the tibial lesion, an FDG/PET was performed for a systemic evaluation. The imaging revealed increased 18FDG uptake in the right proximal tibia (maximum standardized uptake value (SUVmax) 4.90), right proximal clavicle (SUVmax 3.20), and distal humerus (SUVmax 3.20) (Figure 5).

A subsequent incisional biopsy of the right clavicle confirmed that the pathological features were consistent with those of the tibial lesion, leading to a definitive diagnosis of CRMO. Pain remission was achieved after two months of oral non-steroidal anti-inflammatory drug (NSAID) administration, and no pain recurrence was noted during the 14-month follow-up period.

## Discussion

CRMO is an autoimmune bone inflammatory disorder that predominantly affects children and adolescents. First identified in 1972 as chronic abacterial osteomyelitis [1], the prevalence of CRMO in the pediatric population is estimated to be between 0.4 and 2 per 100,000 children [2,3].

CRMO is primarily characterized by non-purulent inflammation and typically presents with multiple bone lesions, with the metaphyseal regions of long bones, clavicles, and vertebral bodies being frequently affected sites. The clinical manifestations include spontaneous bone pain, swelling, tenderness, and restricted movement. Due to the lack of standardized diagnostic criteria and established biomarkers, CRMO is often diagnosed by exclusion, necessitating a comprehensive assessment that integrates clinical evaluation, laboratory findings, imaging studies, and histopathological examinations to rule out other conditions [2-4].

The disease predominantly affects children and adolescents and must be differentiated from malignant bone tumors, such as osteosarcoma and Ewing's sarcoma [5,6]. These malignancies share overlapping features with CRMO, including similar onset age and a predilection for long bones. Additionally, both CRMO and Ewing's sarcoma commonly present with elevated inflammatory markers, such as CRP and erythrocyte sedimentation rate, complicating differentiation based solely on imaging. Consequently, histopathological analysis is often necessary to exclude malignancies [7-11].

Imaging studies are essential for diagnosing CRMO, with commonly employed modalities including CT, MRI, and radionuclide bone scintigraphy. CT is effective in identifying specific CRMO lesions. However, its ability to detect early asymptomatic lesions is limited due to the disease's multifocal nature [12]. Whole-body MRI, considered the gold standard for detecting both symptomatic and asymptomatic lesions, is invaluable in pediatric CRMO diagnosis and monitoring. It excels at identifying bone marrow edema, an indicator of inflammation, thereby enhancing diagnostic sensitivity [13]. However, MRI findings are not specific and may resemble conditions such as infections, Langerhans cell histiocytosis, and malignant tumors like Ewing's sarcoma and osteosarcoma. Additionally, the need for sedation or anesthesia in younger children can limit the feasibility of MRI.

FDG/PET is useful for differentiating malignant from benign lesions based on metabolic activity, as malignant tumors generally exhibit high FDG uptake while benign lesions, including those in CRMO, show low uptake. However, the inflammatory nature of CRMO lesions means FDG/PET can produce false positives or negatives, necessitating careful interpretation alongside other clinical and imaging findings. While the utility of FDG PET/CT in diagnosing and monitoring chronic inflammatory lesions in children has been demonstrated in previous studies, its specific application in evaluating CRMO remains to be clearly established [14-16].

A recent series of case studies by Xu et al. examined the diagnostic effectiveness of FDG PET/CT in CRMO patients [17]. They analyzed data from 21 patients, averaging 9.4 years in age, and found that each patient exhibited more than six lesions on average (range: 2-14), with 19.0% of patients displaying more than ten lesions. The most commonly affected areas were the tibia or fibula (66.7% of patients), followed by the femur (52.4%), pelvis (47.6%), and feet (42.9%). The recorded SUVmax values ranged from 1.2 to 10.9. These observations align with our case findings, where the tibia showed significant uptake with a maximum SUVmax of 4.90. In contrast, the elevated SUVmax in the clavicle and humerus is considered atypical, indicating that such non-specific clinical and imaging findings may pose challenges for the definitive diagnosis of CRMO. Furthermore, elevated SUVmax values are not specific to inflammatory diseases. Stephan et al. performed PET/CT imaging on 77 patients with liver conditions and measured the SUVmax in both malignant tumors and inflammatory/infectious lesions. Their findings revealed no significant differences in SUVmax between these two conditions [18]. This result highlights the challenge in distinguishing between inflammatory and malignant diseases based solely on PET/CT imaging.

The treatment approach for CRMO is not yet standardized, aiming primarily at alleviating pain and ensuring proper bone development. NSAIDs are the preferred initial therapy; however, they can frequently result in disease recurrence in many patients [19]. When first-line treatment with NSAIDs fails or is inadequate, second-line options, such as corticosteroids, TNF-αinhibitors, DMARDs, and bisphosphonates, are employed [20]. Bisphosphonates, known for their strong inhibition of osteoclast-driven bone resorption, have been demonstrated to be effective in managing CRMO.

If left untreated, CRMO can result in significant complications, such as spinal fractures and kyphosis, which may lead to neurological impairments and motor dysfunction, as well as considerable psychological distress [4]. Moreover, distinguishing CRMO from malignant bone tumors like osteosarcoma and Ewing's sarcoma is crucial, as delayed diagnosis of these malignancies can adversely affect survival outcomes, underscoring the need for timely diagnosis and intervention.

In conclusion, while FDG PET/CT offers valuable insights into distinguishing CRMO from other conditions, an incisional biopsy remains crucial for a definitive diagnosis, especially when early confirmation is imperative. We have discussed the utility of FDG/PET in diagnosing CRMO based on a single case; hence, our experience is limited. Consequently, we cannot fully assess its diagnostic utility for CRMO. Future studies involving larger cohorts are necessary to evaluate the significance of FDG/PET in this context. Additionally, further research should focus on developing reliable biomarkers and refining imaging modalities to enhance early diagnosis and management of CRMO, thereby improving patient outcomes.

## Conclusions

This case highlights the diagnostic challenges associated with CRMO, particularly in distinguishing it from malignant bone tumors using imaging techniques like FDG-PET. The elevated SUVmax values observed in this case, which are typically associated with malignancies, underscore the limited utility of FDG-PET in differentiating CRMO from conditions such as osteosarcoma and Ewing's sarcoma. Despite the benign nature of CRMO, the high FDG uptake may mimic that of malignant lesions, thereby complicating the diagnostic process. Therefore, incisional biopsy remains essential for definitive diagnosis, especially when multiple bone lesions are present. This case also reinforces the importance of considering CRMO in the differential diagnosis of pediatric patients presenting with bone lesions, even when imaging findings suggest malignancy.

## References

[1] Catalano-Pons C, Comte A, Wipff J (2008). Clinical outcome in children with chronic recurrent multifocal osteomyelitis. Rheumatology (Oxford).

[2] Hedrich CM, Hahn G, Girschick HJ, Morbach H (2013). A clinical and pathomechanistic profile of chronic nonbacterial osteomyelitis/chronic recurrent multifocal osteomyelitis and challenges facing the field. Expert Rev Clin Immunol.

[3] Nuruzzaman F, Zhao Y, Ferguson PJ (2021). Chronic nonbacterial osteomyelitis: insights into pathogenesis, assessment, and treatment. Rheum Dis Clin North Am.

[4] Hofmann SR, Kubasch AS, Range U, Laass MW, Morbach H, Girschick HJ, Hedrich CM (2016). Serum biomarkers for the diagnosis and monitoring of chronic recurrent multifocal osteomyelitis (CRMO). Rheumatol Int.

[5] Nepal P, Alam SI, Sajid S, Sapire J, Ojili V (2021). Rare presentation of chronic recurrent multifocal osteomyelitis of the Iliac wing mimicking Ewing's sarcoma. SA J Radiol.

[6] Figueiredo MP, Pato M, Amaral F (2017). Chronic recurrent multifocal osteomyelitis: a case report with atypical presentation. J Orthop Case Rep.

[7] Zhang P, Jia XY, Zhang Y, Morelli J, Zhang ZK (2017). Chronic recurrent multifocal osteomyelitis beginning with a solitary lesion of the ilium. BMC Musculoskelet Disord.

[8] Gicchino MF, Diplomatico M, Granato C, Capalbo D, Marzuillo P, Olivieri AN, Miraglia Del Giudice E (2018). Chronic recurrent multifocal osteomyelitis: a case report. Ital J Pediatr.

[9] Huang E, Wolfe VG, Yaeger SK, Fugok KL (2023). Case presentation of a nine-year-old female with chronic recurrent multifocal osteomyelitis. Cureus.

[10] Sgaglione J, Muran A, Rhode M (2024). Geriatric chronic recurrent multifocal osteomyelitis (CRMO) mimicking multifocal multiple myeloma: a first in an octogenarian. Skeletal Radiol.

[11] Newman EN, Jones RL, Hawkins DS (2013). An evaluation of [F-18]-fluorodeoxy-D-glucose positron emission tomography, bone scan, and bone marrow aspiration/biopsy as staging investigations in Ewing sarcoma. Pediatr Blood Cancer.

[12] Costa-Reis P, Sullivan KE (2013). Chronic recurrent multifocal osteomyelitis. J Clin Immunol.

[13] Andronikou S, Kraft JK, Offiah AC (2020). Whole-body MRI in the diagnosis of paediatric CNO/CRMO. Rheumatology (Oxford).

[14] Signore A, Glaudemans AW, Gheysens O, Lauri C, Catalano OA (2017). Nuclear medicine imaging in pediatric infection or chronic inflammatory diseases. Semin Nucl Med.

[15] Liu Y (2016). Chronic nonbacterial osteomyelitis with FDG avid rib destruction and extensive lymphadenopathy. Clin Nucl Med.

[16] Hong YH (2013). Three cases of fever of unknown origin (FUO) with acute multifocal non-bacterial osteitis (NBO) as reactive osteomyelitis. Rheumatol Int.

[17] Xu Y, Wang G, Wang Y, Wang W, Kan Y, Yang J (2024). Diagnostic role of FDG PET/CT in pediatric patients with chronic recurrent multifocal osteomyelitis. Clin Nucl Med.

[18] Skawran S, Messerli M, Kotasidis F (2022). Can dynamic whole-body FDG PET imaging differentiate between malignant and inflammatory lesions?. Life (Basel).

[19] Wipff J, Costantino F, Lemelle I (2015). A large national cohort of French patients with chronic recurrent multifocal osteitis. Arthritis Rheumatol.

[20] Eleftheriou D, Gerschman T, Sebire N, Woo P, Pilkington CA, Brogan PA (2010). Biologic therapy in refractory chronic non-bacterial osteomyelitis of childhood. Rheumatology (Oxford).

